# How does internet use affect the mental health of older adults: examining the mediating pathways of perception of social fairness and physical activity

**DOI:** 10.3389/fpubh.2026.1765599

**Published:** 2026-02-11

**Authors:** Fuxiang Yu, Long Niu, Yifei Shen

**Affiliations:** 1 Business School, Hangzhou City University, Hangzhou, China; 2 School of Physical Education, Xi'an Jiaotong University, Xi'an, China; 3 Department of Physical Education, Xidian University, Xi'an, China

**Keywords:** internet use, mental health, older adults, perception of social fairness, physical activity

## Abstract

**Background:**

The association between internet use and the mental health of older adults presents a complex research issue with inconsistent findings. While potential mechanisms have been explored, the integrated examination of socio-cognitive and health-behavioral pathways, particularly the chained mediation effect, remains understudied.

**Methods:**

Utilizing cross-sectional data from the 2023 China General Social Survey (CGSS), this study analyzed a sample of 3,206 adults aged 60 and above. Mental health was operationalized using a single-item measure of the frequency of depressive moods. A structural equation model (SEM) was employed to explore the parallel and chained mediating roles of perceived social justice and physical activity in the relationship between internet use and mental health. It should be acknowledged that these mediation analyses are statistical and exploratory in nature, and do not establish temporal or causal ordering. Robustness checks were conducted using propensity score matching (PSM).

**Results:**

Internet use was significantly and directly associated with better mental health (*β* = 0.098, *p* < 0.001). Parallel mediation analysis revealed two significant but opposing indirect paths: a negative indirect effect through reduced perceived social justice (*β* = −0.017, *p* < 0.001) and a positive indirect effect through increased physical activity (*β* = 0.027, *p* < 0.001). Crucially, a significant chained mediation pathway was identified: internet use → lower perceived social justice → reduced physical activity → poorer mental health (*β* = −0.018, *p* < 0.001). Multi-group analysis confirmed significant gender differences in these mediating pathways.

**Conclusion:**

Internet use is associated with older adults’ mental health through a complex mechanism involving both a direct positive association and indirect pathways via perceived social justice and physical activity. The verified chained mediation effect suggests a potential “cognition-behavior” transmission pathway. A key limitation of this study is its cross-sectional design, which precludes causal inference. These findings highlight the dual-edged nature of internet use, suggesting that public health initiatives aimed at promoting digital inclusion for aging populations could concurrently foster a positive online information environment, enhance digital literacy, and encourage physical activity to maximize benefits and mitigate risks.

## Introduction

The global aging population has increasingly highlighted the importance of mental health among older adults as a public health priority (1). There is growing recognition that mental health constitutes a vital dimension of overall quality of life for the older adults. Aging is a natural life stage characterized by cumulative changes across physical, psychological, and social dimensions, underscoring the necessity of multidimensional strategies to maintain and enhance quality of life. Key elements include emotional wellbeing, social engagement, physical health, and functional independence—collectively promoting active and healthy aging. Among evidence-based non-pharmacological interventions, physical exercise is consistently recommended to improve older adults’ quality of life (2), while social participation and cognitive stimulation also play indispensable roles (3).

In this context, internet use presents a double-edged sword, simultaneously promoting mental health and overall quality of life while posing distinct risks (4). Some studies indicate that internet use can alleviate loneliness and strengthen social connections (5), thereby promoting social engagement. However, other research suggests that upward social comparison and information overload may lead to negative outcomes (6), including reduced perceptions of social fairness (7). These conflicting findings underscore the importance of investigating how digital engagement influences multidimensional quality of life in later life and how it can be optimized to support it. To unravel these complex relationships, it is essential to explore mediating mechanisms beyond direct effects. Existing research has examined only isolated pathways involving cognitive factors (e.g., perceived social justice) or behavioral factors (e.g., physical activity) (8, 9). Concurrently, physical activity interventions must account for sociodemographic variations and adapt to older adults’ diverse characteristics, interests, and resources to promote equitable and inclusive aging.

This study focuses on perceived social fairness—defined as individuals’ subjective evaluations of equitable resource distribution—as a key cognitive mediator. This construct is chosen due to its established link to mental health (10), responsiveness to online information exposure, and the availability of validated measurement tools in the China General Social Survey (CGSS). Cognitive-behavioral theory provides an effective framework for elucidating how internet use influences mental health and quality of life through sequential cognitive and behavioral pathways (11). This theory posits that cognitive evaluations (e.g., perceived social justice) shape behavioral choices (e.g., participation in physical activities), which in turn influence psychological outcomes (12). This perspective suggests that internet use may be associated with mental health through a chain of mediating pathways involving perceptions of social fairness and physical activity, both core elements of positive aging.

Based on this theoretical framework, this study aims to: (1) Examine the association between older adults’ internet use and mental health; (2) investigate the mediating role of perceived social fairness and physical activity; (3) test whether these variables exert effects through sequential mediating pathways. By achieving these objectives, this study aims to deepen understanding of the complex relationship between internet use in later life and mental health, providing evidence for interventions promoting digital engagement, physical activity, and social participation to support enhanced quality of life and positive aging.

## Literature review and research hypotheses

### Internet use and mental health in older adults

Against the backdrop of active aging strategies, the mechanisms influencing the mental health of the older adults have become a core issue of concern for both academia and policymakers. In traditional research, due to the generally slower adoption of emerging technologies among the older adults, the impact of digital technology factors on their mental health had long been underexplored. With the acceleration of digitalization, age-friendly adaptations in internet applications have significantly lowered the barriers to technology use, leading to a continuous expansion of the older adults internet user population. Data show that in 2024, the internet usage rate among adults aged 65 and above in the United States had reached 90%, while the internet penetration rate among those aged 60 and above in China also reached 52.95% during the same period (13). In this context, the relationship between internet use and mental health in older adults has gradually become a research focus. Early studies, often based on small-sample surveys, found that internet use had a positive effect on the mental health of the older adults, but the generalizability of these conclusions was limited (14, 15). With the increasing availability of large-scale micro-level survey data, subsequent research has been able to more systematically uncover the complex relationship between the two. A study by Cotten et al. (16) based on U.S. data showed that internet use significantly reduced depression levels among retired older adults individuals. Lelkes, using data from multiple European countries, demonstrated that the internet enhanced subjective wellbeing among the older adults by alleviating loneliness and strengthening social connections (17). Furthermore, a longitudinal study found that the frequency of internet use among Japanese older adults was significantly positively correlated with their health status and social wellbeing 3 years later (18).

However, the academic community has not yet reached a consensus regarding the relationship between internet use and the health of older adults. Some studies indicate that there is no significant correlation between the two; on the contrary, issues such as the digital divide, information overload, or internet addiction may even negatively impact the health of older adults (19, 20). Despite these controversies, most scholars agree that the effects of internet use are primarily mediated through intermediary mechanisms, with its positive influence manifesting across multiple dimensions such as social participation, social support, and self-efficacy (21, 22). In recent years, research in this field has developed rapidly. A growing body of evidence suggests that internet use has a robust promotive effect on the mental health of middle-aged and older adults (23). However, its impact is significantly moderated by factors such as usage patterns, online functions, and user group characteristics. On one hand, the use of social and educational applications has shown more pronounced benefits for mental health, whereas the influence of entertainment-oriented functions appears weaker. Moreover, urban and younger older adults populations tend to derive greater benefits (24). On the other hand, internet use demonstrates more substantial health benefits for older adults with higher education levels, those without a spouse, and individuals with a political background (e.g., party membership), with studies highlighting the crucial role of leisure-oriented applications (25). In contrast, some research suggests that populations with lower education levels may experience higher marginal gains from using new media (26). Additionally, certain studies also caution that excessive internet use may adversely affect health through mechanisms such as reduced sleep duration (27).

The aforementioned studies indicate that the impact of internet use on the mental health of older adults exhibits multidimensional and heterogeneous characteristics. According to the Selective Optimization with Compensation (SOC) theory, as older adults face declines in physiological function and social roles, they can utilize internet technology to reconstruct patterns of resource acquisition and social connection. By optimizing behavioral choices, they compensate for their own limitations, thereby maintaining psychological wellbeing (28). Especially in the context of continuous advancements in age-friendly digital adaptations, social media and digital platforms provide new pathways for older adults to achieve social re-engagement and enhance their sense of control over life (29). Based on this, we propose the following Hypothesis 1:

*H1*: Internet use has a significant positive impact on the mental health of older adults.

### The mediating role of sense of social fairness

Furthermore, theoretical perspectives from social psychology, such as social comparison theory (30) and relative deprivation theory (31), provide critical frameworks for understanding the potential psychological impacts—both positive and negative—of internet adoption among older adults. These theories suggest that online environments can fundamentally alter individuals’ reference groups and amplify perceptions of inequality or injustice, mechanisms which may be particularly salient for older populations navigating increasingly digital social spaces. Perception of social fairness refers to an individual’s subjective recognition and judgment of whether the distribution of social resources and opportunities is just. It serves as an important predictor for measuring social mentality and psychological wellbeing (32). Against the backdrop of an aging society and digital convergence, internet usage has become a key external factor influencing older adults’ social cognition and psychological state. On one hand, as a major channel for information acquisition and social interaction, the internet can enhance older adults’ perception of social fairness by increasing information transparency, broadening social participation, and improving resource accessibility (33). On the other hand, the internet may also negatively affect this perception through mechanisms such as exposure to negative news, upward social comparison (34), relative deprivation (35), or exacerbated digital inequality (36). This dual potential positions the perception of social fairness as a likely mediating factor between internet use and mental health among older adults.

According to the cognitive appraisal theory, an individual’s cognitive evaluation of the external environment significantly influences their emotional state and psychological adaptability (37). As a technological behavior, internet use not only transforms older adults’ lifestyles but also indirectly shapes their judgment of social fairness by altering their information environment and social reference systems. When older adults perceive society as more equitable, their psychological stress is alleviated and their sense of self-worth is enhanced, thereby contributing to improved overall mental health. Conversely, a lower perception of social fairness may lead to negative emotions such as alienation and helplessness, which in turn suppress psychological wellbeing (38). Therefore, the perception of social fairness not only reflects older adults’ cognitive feedback regarding the broader social environment but also constitutes an intrinsic mechanism shaping their psychological state. Based on this, the study proposes Hypothesis 2:

*H2*: Perceived social fairness mediates the relationship between internet use and mental health among older adults.

### The mediating role of physical activity

In exploring the complex relationship between internet use and mental health among older adults, physical activity is increasingly regarded as a potential key mediating variable. The establishment of this mediating pathway stems from the potential positive impact of internet use on the level of physical activity among older adults, as well as the well-documented significant role of physical activity in promoting psychological wellbeing. From the perspective of the mechanisms through which internet use influences physical activity, digital technology provides an unprecedented support system for older adults to engage in physical activity. Research indicates that the internet effectively lowers barriers to participation in physical activity through convenient access to health information (39), the availability of online fitness courses (40), and social support within virtual exercise communities (41). Together, these technological features create a favorable environment that encourages engagement in physical activity.

Furthermore, the psychological benefits of physical activity have been well-documented. Physiological studies indicate that regular physical activity can improve mental states through mechanisms such as regulating the neuroendocrine system (42) and promoting the secretion of brain-derived neurotrophic factor (43). From a psychosocial perspective, physical activity also enhances mental health by boosting self-efficacy, increasing social interaction, and strengthening one’s sense of meaning in life (44). Although both of these pathways have received substantial empirical support, research on the mediating role of physical activity between internet use and mental health remains limited. Most existing literature examines either the impact of internet use on physical activity or the effect of physical activity on mental health in isolation, lacking comprehensive empirical examination of the full mediating pathway. This gap in the literature hinders a deeper understanding of how digital technology influences older adults’ psychological wellbeing through behavioral mechanisms. Based on this, the study proposes Hypothesis 3:

*H3*: Physical activity mediates the relationship between internet use and mental health among older adults.

### The chain mediating effect of sense of social fairness and physical activity

As a significant social resource and information medium, internet use not only directly influences the mental health of older adults but may also trigger psychosocial mechanisms and promote healthy behaviors, forming a multi-level chain mediation pathway. Among these mechanisms, the sense of social fairness—referring to an individual’s subjective perception of justice in the distribution of social resources—and physical activity, as a health-promoting behavior, together constitute intrinsic pathways through which internet use affects older adults’ psychological wellbeing. Specifically, internet use enhances older adults’ sense of social fairness through multiple channels: on the one hand, digital access improves their ability to obtain information and opportunities for social participation, thereby reducing the sense of inequity caused by information asymmetry (45); on the other hand, social media and remote interaction help maintain intergenerational communication and expand social networks, which can alleviate feelings of social isolation and strengthen their sense of agency and recognition in resource distribution (46). An enhanced sense of social fairness may not only directly contribute to psychological wellbeing but also further encourage the adoption of healthy behaviors. According to self-determination theory, when individuals perceive their social environment as supportive and fair, their intrinsic motivation and autonomy increase significantly, making them more inclined to adopt beneficial health behaviors such as regular physical activity (47).

**Figure 1:**
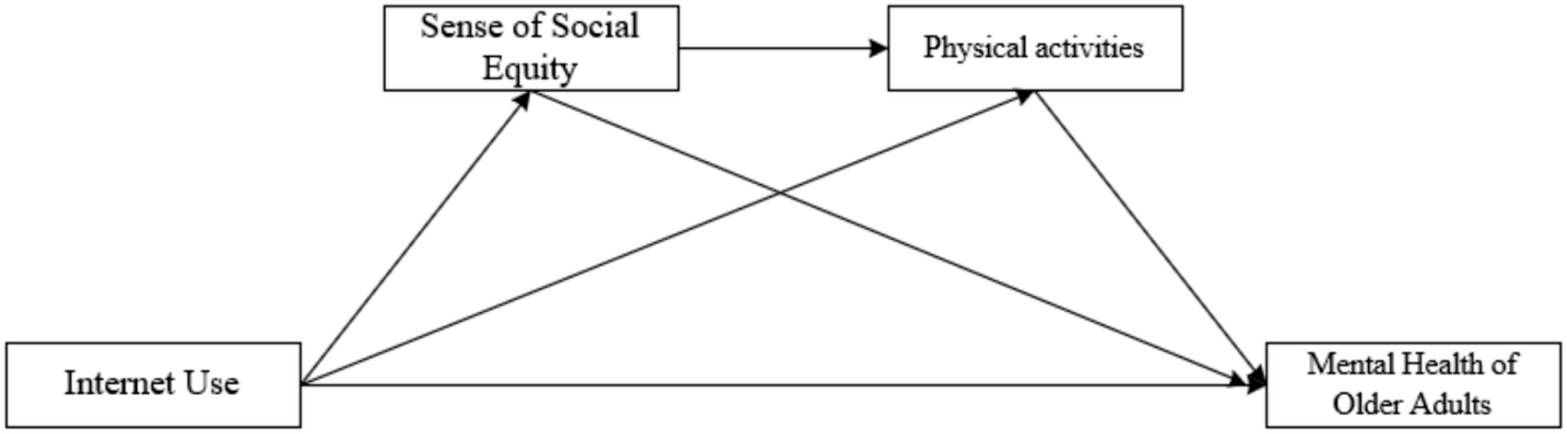
Conceptual model of chain-based intermediation.

Furthermore, a close theoretical connection exists between the sense of social fairness and engagement in physical activity. Research indicates that individuals who perceive a higher level of social fairness are more likely to develop positive self-perceptions and a greater sense of control, which in turn increases their willingness to participate in group physical activities and public health programs (48). Adherence to Physical activity not only benefits physical and mental health but also reinforces one’s sense of social integration and institutional trust, creating a positive feedback loop (49). Although existing studies have separately demonstrated the role of internet use in promoting social fairness—such as by bridging the digital divide and enhancing social participation—as well as the influence of social fairness on physical activity and mental health among older adults, no research has yet integrated these three elements into a unified analytical framework to examine their chain mediating mechanism in the relationship between internet use and psychological wellbeing. In particular, there is a lack of empirical investigation into the psychosocial and behavioral pathways specific to older adult populations. Although the chain mediation pathway involving perceived social fairness and physical activity has not been extensively tested empirically, the proposed model is grounded in well-established theoretical frameworks, including Self-Determination Theory and Cognitive Appraisal Theory. Therefore, Hypothesis 4 is theoretically derived rather than purely exploratory. Based on the above theoretical reasoning and empirical gaps, this study constructs a chain mediation conceptual model (see Figure 1) and proposes Hypothesis 4:

*H4*: Perceived social fairness and physical activity sequentially mediate the relationship between internet use and mental health among older adults.

## Data, variables, and methodology

### Data sources

This study utilizes data from the 2023 China General Social Survey (CGSS), a nationally representative social survey project that employs multistage stratified probability sampling to collect multidimensional social data. The CGSS is particularly suitable for examining mental health and internet use among older adults due to its comprehensive coverage of psychosocial variables, its inclusion of a substantial sub-sample of respondents aged 60 and above, and its incorporation of validated measures specifically relevant to aging populations, such as subjective wellbeing, social justice perceptions, and digital engagement behaviors. The survey is widely recognized as an authoritative data source in social science research in China, offering extensive geographic coverage spanning 31 provincial-level administrative regions and maintaining a balanced representation of both urban (52.4%) and rural (47.6%) residences. A key feature of the CGSS is its use of concise single-item measures, which serve as validated proxies for complex constructs (e.g., mental health) and are suitable for capturing population-level trends.

The analysis focused on respondents aged 60 and above (*n* = 3,812 initially identified). The sample screening process involved multiple steps: first, we excluded respondents with missing values on core study variables (*n* = 406); second, we implemented logical checks to identify and remove inconsistent responses (*n* = 112); third, we applied Tukey’s method to detect and winsorize extreme outliers (*n* = 88) at 3 standard deviations from the mean for continuous variables. The final analytical sample consisted of 3,206 individuals, representing an 84.1% retention rate of the original older adults sub-sample. Survey weights were used to ensure representativeness. The sample showed balanced gender distribution (51.81% male, 48.19% female) and adequate age group representation: 60–69 years (52.3%), 70–79 years (38.1%), and 80+ years (9.6%). As a secondary analysis of anonymized, publicly available data, this study required no additional ethical approval.

### Variable measurement

The variables described below were operationalized to specifically test the hypotheses outlined in this study (H1–H4), which propose relationships between Internet Use, Mental Health, and the mediating roles of Perceived Social Fairness and Physical activity.

*Dependent variable: mental health*. The dependent variable in this study is mental health, which is operationally defined as the frequency of depressive mood recently experienced by respondents. This ordinal categorical variable was measured using the question from the CGSS 2023 questionnaire: “In the past 4 weeks, how often have you felt depressed or down?” The original item includes five response categories: 1 = “Never,” 2 = “Rarely,” 3 = “Sometimes,” 4 = “Often,” and 5 = “Always.” In the analysis, higher values on this variable indicate more frequent occurrences of depressive mood, reflecting poorer mental health status, while lower values correspond to more positive psychological states. This single-item measure, while concise, is a well-validated proxy for general psychological distress in large-scale surveys (50). It strongly correlates with established multi-item scales (e.g., CES-D) and effectively captures population-level mental health gradients (51). We therefore interpret it as a indicator of overall mental health status, with its focus on core affective experience.

*Independent variable: internet usage*. This ordinal variable, ranging from 1 to 5, captures the frequency of respondents’ internet usage (including via mobile phones), with values corresponding to increasing levels of use from never to very frequent. It is derived from the CGSS 2023 survey question: “In the past year, how often have you used the following media—the Internet (accessing the web via mobile phones, computers, tablets, smart wearable devices, etc.)?” with response options including 1 = “Never,” 2 = “Rarely,” 3 = “Sometimes,” 4 = “Often,” and 5 = “Very frequently.” It is important to note that this measure captures the frequency of general internet use but does not distinguish between different types (e.g., social, informational, recreational) or purposes of use. While frequency is a fundamental and widely used dimension in digital inequality research, this limitation means our findings speak to the association between how often older adults go online and their mental health, rather than the specific nature of their online activities.

*Mediating variables: perceived social fairness and physical activity*. This study employs perceived social fairness and physical activity frequency as mediating variables. Perceived social fairness measures individuals’ subjective judgments regarding the equitable distribution of societal resources and opportunities. This variable originates from the 2023 China General Social Survey (CGSS) question: “Overall, do you think society is fair today?” “using a five-point Likert scale: 1 = Completely unfair, 2 = Somewhat unfair, 3 = Neutral, 4 = Somewhat fair, 5 = Completely fair. Higher scores indicate more positive evaluations of social fairness. This construct differs from social trust (measuring belief in the integrity of others or institutions) and subjective social class (measuring individuals’ self-perceived position in the social hierarchy). Perceived social fairness specifically captures evaluations of social rules—whether societal systems are just. Its unique significance for mental health (especially in later life) lies in the belief that living in a just world (where effort yields fair rewards) has the potential to buffer stress, thereby reducing feelings of helplessness and resentment.

Physical activity reflects older adults’ patterns of participation in sports activities, measured using the CGSS 2023 scale item: “How often did you participate in sports activities during your leisure time in the past year?” Response options: 1 = “Never participated,” 2 = “Participated a few times a year or less,” 3 = “Participated a few times a month,” 4 = “Participated a few times a week,” 5 = “Participated daily.” Higher values indicate greater frequency of physical activity participation. The terms “leisure time” and “physical activity” in this item indicate it encompasses voluntary, conscious physical exertion, including both structured activities (e.g., gym workouts, Tai Chi classes) and unstructured activities (e.g., walking, gardening). While this broad interpretation helps comprehensively capture active leisure behaviors, it means we cannot separately assess the specific impact of high-intensity structured exercise. The findings of this study should be interpreted as indicating a beneficial association between older adults’ participation in any form of regular leisure-time physical activity and their mental health.

*Mediating variables: perception of social fairness and physical activity*. This study incorporates perception of social fairness and frequency of Physical activity as mediating variables. The perception of social fairness is used to measure individuals’ subjective judgment regarding the fairness of resource and opportunity distribution in society. This variable is derived from the 2023 Chinese General Social Survey (CGSS) question: “Overall, how fair do you think society is today?” which employs a five-point Likert scale: 1 = “Completely unfair,” 2 = “Somewhat unfair,” 3 = “Neutral,” 4 = “Somewhat fair,” and 5 = “Completely fair.”

Higher scores indicate a more positive evaluation of social fairness. Physical activity reflects the behavioral patterns of older adults engaging in sports activities, measured using the CGSS 2023 item: “In the past year, how often did you engage in Physical activity during your leisure time?” with response options: 1 = “Never,” 2 = “A few times a year or less,” 3 = “A few times a month,” 4 = “A few times a week,” and 5 = “Every day.” Higher values represent a greater frequency of participation in Physical activity.

*Control variables*: To isolate the net relationships between the core variables and mitigate potential confounding effects, this study incorporates a set of control variables encompassing fundamental demographic and socioeconomic characteristics. These include: gender (0 = male, 1 = female), type of residence (0 = rural, 1 = urban), age (continuous, measured in actual years), marital status (0 = not currently married, 1 = married or cohabiting), subjective socioeconomic status (self-reported categorization as 1 = lower class, 2 = middle class, 3 = upper class), educational attainment (ordinal, 1 = no formal education to 6 = postgraduate or above), and self-rated health status (1 = very unhealthy to 5 = very healthy).

### Analytical methods

This study employs a multistage analytical approach to test the hypothesized relationships. First, descriptive statistics and correlation analysis were used to characterize the sample distribution and assess the direction and strength of associations among core variables. Second, structural equation modeling (SEM) was applied to analyze mediating pathways, examining the mediating effects of perceived social fairness and physical activity (as independent and sequential mediators, respectively) between internet usage and mental health. In the SEM analysis, following methodological recommendations (i.e., treating Likert-scale variables as continuous when five or more variables are present and the sample size is large), Likert-scale variables were treated as continuous (52). The statistical significance of mediating effects was assessed using a guided resampling procedure with 1,000 iterations. Model fit indices CFI > 0.95 and RMSEA < 0.05 indicated good model fit. To ensure robustness, alternative model specifications (including reversing the order of mediating variables) were tested. Third, to explore potential gender differences in these mediating paths, a multi-group structural equation model (SEM) was employed to analyze the sample stratified by gender (male vs. female). Path coefficients across groups were compared using likelihood ratio tests. Finally, to eliminate self-selection bias from observed confounders, the average treatment effect (ATT) by dichotomizing network usage and balancing covariates via nearest-neighbor matching with a 0.02 caliper width. It should be noted that while propensity score matching mitigates observed variable bias, it cannot control unobservable confounders nor fully establish causality. All analyses were conducted using Stata 17.0 software.

## Results

### Descriptive statistical analysis

Descriptive statistics were conducted for all variables in the study, with results summarized in Table 1. The analytical sample consisted of 3,206 older adults. Regarding core variables, the mean score for mental health was 3.066 (SD = 1.109). Internet use frequency averaged 2.555 (SD = 1.629), indicating a moderate to low level of engagement. Perceived social fairness had a mean score of 3.462 (SD = 1.028), slightly above the theoretical midpoint. Physical activity frequency averaged 2.321 (SD = 1.702), suggesting generally low levels of physical activity among the older adult population. Among control variables, the average age of respondents was 70.68 years (SD = 7.019). Gender distribution was relatively balanced, with males accounting for 51.8% of the sample. The majority of participants resided in urban areas (60.3%) and were married or cohabiting (72.8%). Subjective socioeconomic status was concentrated in the middle-lower range (M = 1.505, SD = 0.628), and educational attainment was predominantly at the junior high school level or below (M = 2.919, SD = 1.247). All variable values fell within predefined measurement ranges, with no extreme outliers detected, confirming that data quality met analytical requirements.

**Table 1:** Descriptive statistics of variables.

Variable	*N*	Mean	SD	Min	Max
Mental health	3,206	3.066	1.109	1	5
Internet use	3,206	2.555	1.629	1	5
Perceived social fairness	3,206	3.462	1.028	1	5
Physical exercise	3,206	2.321	1.702	1	5
Gender	3,206	1.482	0.500	1	2
Age	3,206	70.676	7.019	61	96
Type of residence	3,206	0.603	0.489	0	1
Educational attainment	3,206	2.919	1.247	1	6
Self-rated health	3,206	3.065	1.109	1	5
Marital status	3,206	0.728	0.445	0	1
Socioeconomic status	3,206	1.505	0.628	1	3

### Pearson correlation analysis

Table 2 presents Pearson correlation analyses examining the relationships among mental health, internet usage, perceived social fairness, and physical activity. The results indicate that mental health among older adults was significantly and positively correlated with internet use (*r* = 0.108, *p* < 0.001) and Physical activity (*r* = 0.231, *p* < 0.001), but significantly and negatively correlated with perceived social fairness (*r* = −0.129, *p* < 0.01). Perceived social fairness showed a significant positive correlation with mental health (*r* = 0.115, *p* < 0.001), yet only a weak positive correlation with Physical activity (*r* = 0.041, *p* < 0.05). Physical activity was also significantly and positively correlated with mental health (*r* = 0.134, *p* < 0.001). These findings preliminarily suggest that internet use, perceived social fairness, and physical activity are all closely associated with the mental health of older adults, providing a statistical foundation for subsequent analyses.

**Table 2:** Correlation matrix of key variables.

Variable	Mental health	Internet use	Perceived social fairness	Physical activity
Mental health	1.000			
Internet use	0.108^***^	1.000		
Perceived social fairness	0.115^***^	−0.129^***^	1.000	
Physical activity	0.134^***^	0.231^***^	0.041^***^	1.000

^***^p < 0.01.

### Mediation analysis

To examine the internal mechanism through which internet use affects the mental health of older adults, this study constructed a parallel mediation model incorporating perceived social fairness and physical activity. All reported coefficients are standardized unless otherwise specified. The model fit indices demonstrated good alignment with the data: CFI = 0.958, TLI = 0.951, RMSEA = 0.042, SRMR = 0.036, all meeting established thresholds for acceptable model fit, thus supporting the reliability of the structural equation modeling (SEM) results. The analytical results (see Table 3) indicate that internet use has a significant positive total effect on the mental health of older adults (*β* = 0.108, 95% CI [0.086, 0.130]). Further path analysis revealed that this influence is realized through the following three mechanisms:

**Table 3:** Parallel mediation model analysis of the impact of internet use on mental health (*N* = 3,206).

Path and effect	Coefficient	Std. error	z-value	*p*-value	95% CI
Total effect	0.108	0.011	6.12	<0.001	[0.086,0.130]
Direct path					
Internet use → mental health	0.098	0.012	5.70	<0.001	[0.044, 0.090]
Indirect paths					
a: Internet use → perceived social fairness	−0.129	0.011	−7.31	<0.001	[−0.103, −0.060]
b: Perceived social fairness → mental health	0.132	0.019	7.36	<0.001	[0.104, 0.180]
c: Internet use → physical activity	0.229	0.018	13.06	<0.001	[0.204, 0.276]
d: Physical activity → mental health	0.116	0.011	6.84	<0.001	[0.054, 0.098]
e: Perceived social fairness → physical activity	0.011	0.029	5.63	<0.001	[0.039, 0.075]
Effect decomposition					
Direct effect	0.098	0.012	5.70	<0.001	[0.044, 0.090]
Indirect effect 1 (a × b)	−0.017	0.002	−5.32	<0.001	[−0.016, −0.007]
Indirect effect 2 (c × d)	0.027	0.003	6.23	<0.001	[0.012, 0.024]
Chain mediation effect (a × e × d)	0.018	0.002	5.62	<0.001	[0.032, 0.055]

All coefficients presented are standardized.

First, internet use demonstrates a significant direct promoting effect on the mental health of older adults (*β* = 0.098, *p* < 0.001, 95% CI [0.044, 0.090]), supporting Hypothesis H1. This suggests that, after controlling for all mediating paths and covariates, internet use itself remains an important factor enhancing mental health levels among older adults. This effect may stem from immediate information access, social fulfillment, and entertainment experiences, among other factors.

Second, the study identified two significant independent mediating pathways. On one hand, internet use significantly negatively predicts perceived social fairness (path a: *β* = −0.129, *p* < 0.001, 95% CI [−0.103, −0.060]), and lower levels of perceived social fairness subsequently inhibit mental health (path b: *β* = 0.132, *p* < 0.001, 95% CI [0.104, 0.180]). The resulting indirect effect is negative (a × b: *β* = −0.017, *p* < 0.001, 95% CI [−0.016, −0.007]). This result partially supports Hypothesis H2, confirming that perceived social fairness indeed plays a mediating role, though its direction is contrary to expectations. It suggests that internet use may reduce older adults’ sense of social fairness—possibly through exposure to social comparison or negative information—thereby exerting a slight indirect negative effect on their mental health. On the other hand, internet use significantly promotes engagement in physical activity (path c: *β* = 0.229, *p* < 0.001, 95% CI [0.204, 0.276]), and physical activity, in turn, has a positive effect on mental health (path d: *β* = 0.116, *p* < 0.001, 95% CI [0.054, 0.098]). The resulting indirect effect is significantly positive (c × d: *β* = 0.027, *p* < 0.001, 95% CI [0.012, 0.024]). Thus, Hypothesis H3 is strongly supported, highlighting the positive role of physical activity as a behavioral mechanism in enhancing mental health.

Finally, the study also identified a theoretically meaningful chain mediation pathway. The serial mediation pathway is theoretically plausible as it aligns with the cognitive-behavioral framework, which posits that cognitive appraisals (e.g., perceptions of social fairness) can shape behavioral engagement (e.g., physical activity), which subsequently influences mental health outcomes. Internet use reduces perceived social fairness (path a: *β* = −0.129, *p* < 0.001, 95% CI [−0.103, −0.060]), and decreased perceived social fairness further inhibits participation in physical activity (path e: *β* = 0.011, *p* < 0.001, 95% CI [0.039, 0.075]), ultimately affecting mental health through this chain (path d: *β* = 0.116, *p* < 0.001, 95% CI [0.054, 0.098]). This chain mediation effect (a × e × d) was statistically significant (*β* = 0.018, *p* < 0.001, 95% CI [0.032, 0.055]). Consequently, Hypothesis H4 is validated, indicating that internet use can indirectly influence mental health through a sequential “cognitive–behavioral” transmission mechanism (i.e., perceived social fairness → physical activity). It should be noted that while several indirect and chained mediation effects reached statistical significance (*p* < 0.001), their magnitudes were relatively small in terms of standardized coefficients. This suggests that although these pathways are systematically present in the population, their practical effect sizes remain modest.

### Gender-specific testing

Gender-specific testing Given the potential gender differences in the pathways through which internet use affects mental health, this study conducted gender-specific analyses using multi-group structural equation modeling. Prior to comparing path coefficients across gender groups, measurement invariance was established to ensure that the constructs were measured equivalently between males and females. A sequence of nested models testing configural, metric, and scalar invariance was conducted. The results supported metric invariance (ΔCFI = 0.008, ΔRMSEA = 0.004), indicating that factor loadings were equivalent across groups and thus allowing for meaningful comparison of structural paths. The results (see Table 4) indicate that the mechanism by which internet use influences mental health differs significantly between genders (likelihood ratio test: Δ*χ*^2^(6) = 9.40, *p* = 0.002). Specifically, internet use had a significant direct positive effect on mental health in all genders, but the point estimate was somewhat higher for females (*β* = 0.088) than males (*β* = 0.056), suggesting potential gender differences that warrant further investigation despite the modest effect size difference. In terms of mediating pathways, the negative effect of internet use on perceived social fairness was more pronounced among females (*B* = −0.095) compared to males (*B* = −0.067). Conversely, the positive effect of perceived social fairness on mental health was stronger in males (*B* = 0.171) than in females (*B* = 0.110). Meanwhile, the promoting effect of internet use on physical activity was more substantial among females (*B* = 0.264) than males (*B* = 0.218). However, the beneficial effect of physical activity on mental health was slightly stronger in males (*B* = 0.086) compared to females (*B* = 0.063). Notably, the pathway from perceived social fairness to physical activity reached statistical significance in both groups(males: *B* = 0.049, *p* < 0.001; females: *B* = 0.016, *p* < 0.001), indicating that this mediating mechanism operates in all gender groups.

**Table 4:** Multi-group analysis of the mediation model by gender.

Path	Relationship	Men (*n* = 1,661)			Women (*n* = 1,545)		
		*β*	SE	*p*	*β*	SE	*p*
Direct effect							
	IU → MH	0.056	0.017	0.001	0.088	0.017	<0.001
Indirect effects							
a	IU → PSJ	−0.067	0.016	<0.001	−0.095	0.016	<0.001
b	PSJ → MH	0.171	0.026	<0.001	0.110	0.027	<0.001
c	IU → PA	0.218	0.026	<0.001	0.264	0.026	<0.001
d	PE → MH	0.086	0.016	<0.001	0.063	0.016	<0.001
e	PSJ → PA	0.049	0.040	<0.001	0.016	0.041	<0.001

Model fit: *χ*^2^(0) = 0.00. A likelihood ratio test comparing the constrained model (all paths equal across groups) to the unconstrained model indicated significant differences, Δ*χ*^2^(6) = 9.40, *p* = 0.002. *B*, unstandardized coefficient; *β*, standardized coefficient; SE, standard error; IU, internet use; MH, mental health; PSJ, perceived social justice; PA, physical activity.

### Preference value matching

To address potential self-selection bias in the relationship between internet use and mental health among older adults, this study dichotomized internet usage (non-users = 0, users = 1) to clearly distinguish the intervention and control groups. This approach is theoretically sound as it enables the application of propensity score matching (PSM)—a method requiring a dichotomous intervention variable to estimate the average treatment effect (ATT) of internet adoption. Propensity scores were estimated via a logistic regression model. Controlling for other variables, results (see Table 5) indicate an average treatment effect (ATT) of 0.367 (unstandardized coefficient) for internet use, statistically significant at the 5% level. This finding suggests internet users scored approximately 0.367 points higher on mental health measures than non-users. This result strongly aligns with the core prior conclusion—that internet use significantly promotes mental health. Although PSM cannot fully eliminate all unobservable confounding factors, incorporating key observable confounders enhances the rationale for adequate bias control. After effectively controlling for selection bias arising from observable variables, the findings robustly support the fundamental argument that “internet use positively impacts mental health,” further strengthening the reliability of the study’s primary conclusions.

**Table 5:** Propensity score matching analysis.

Sample	Treated mean	Control mean	Difference (ATT)	Std. error	*t*-value
Before matching	3.167	2.699	0.467***	0.045	10.44
After matching	3.167	2.800	0.367***	0.051	7.23

^***^p < 0.01. Propensity score matching was performed using nearest neighbor 1:1 matching with a caliper. All standardized biases for covariates were below 10% after matching.

## Discussion

Against the backdrop of global aging and digital convergence, this study investigates the association between internet use and the mental health of older adults based on data from the 2023 China General Social Survey (CGSS). While existing research has identified a general relationship between internet use and mental health in this population, the underlying mechanisms remain insufficiently explored. To address this gap, this study examined the potential mediating roles of perceived social fairness and physical activity. The main findings and their implications are discussed below.

First, internet use shows a significant positive association with the mental health of older adults, a result consistent with previous digital aging studies highlighting the adaptive role of technology use in later life (53, 54). From the perspective of socioemotional selectivity theory, the internet offers older adults a means to maintain social ties and emotional fulfillment, supporting socially meaningful engagement (55). Activity theory further suggests that internet use may help compensate for age-related reductions in social roles and physical mobility, thereby contributing to a sense of purpose and daily engagement (56). The internet may thus function not only as an information tool but also as a psychosocial resource that enhances self-efficacy through improved information access and perceived control (57). Applications such as social media and messaging platforms may also support social capital maintenance, potentially alleviating loneliness and depression (58). Additionally, diverse forms of digital entertainment—including videos, games, and interest communities—may offer new channels for emotional regulation and positive experience (59). Compared to studies on younger populations, this research underscores the particular importance of internet use in the aging context, where individuals often face constraints in social support, intergenerational contact, and offline activity. In light of these findings, policy efforts under the framework of active aging and digital inclusion could extend beyond access provision to include digital literacy training, age-friendly interface design, and supportive community and family ecosystems.

Second, the analysis indicates that perceived social fairness and physical activity may serve as mediators in the relationship between internet use and mental health, though their effects differ in direction and magnitude. Internet use was negatively associated with perceived social fairness, which in turn was linked to lower mental health—suggesting a potential indirect negative pathway. This pattern may be partly explained by exposure to online content that emphasizes social comparison, negative news, or inequity, possibly amplifying perceptions of unfairness and reducing trust in societal institutions (60). Such perceptions, as noted in prior work, may foster relative deprivation and anxiety (61). It should be noted, however, that alternative explanations—such as recall bias, cohort effects, or unmeasured confounding—may also contribute to this association. For instance, older adults with preexisting negative social perceptions may use the internet differently or report lower fairness. Conversely, internet use was positively associated with physical activity, which correlated with better mental health—suggesting a compensatory positive pathway. Online resources—such as health information, virtual fitness communities, and exercise tutorials—may help older adults establish and maintain physical activity routines (62). Such digitally supported behaviors may enhance not only physical health but also self-efficacy and social connectedness (63). The mediating effect of physical activity was notably stronger than that of perceived social fairness, indicating that health behavior mechanisms may partly offset the potential psychological risks associated with altered social perceptions. Overall, the results suggest that internet use may relate to mental health through two distinct mediating pathways: one involving perceived social fairness and another involving physical activity. These findings extend theoretical models of digital technology use and psychological outcomes and highlight the practical value of promoting healthy online environments and positive health behaviors in digital inclusion initiatives.

Third, the study identified a sequential pathway: internet use was associated with lower perceived social fairness, which was linked to reduced physical activity, which in turn correlated with poorer mental health. This pattern is consistent with cognitive-behavioral models within digital contexts (64) and suggests that cognitive appraisal (e.g., fairness perceptions) may influence both psychological states and health behaviors. Older adults exposed to comparative or negative online information may develop perceptions of societal unfairness, which could diminish motivation for physical activity—a phenomenon sometimes described as learned helplessness (65). This cascading mechanism aligns with the “cognition–behavior–outcome” model proposed by Marques et al. (66) and underscores that internet use may relate to mental health through multi-stage processes. From an applied perspective, these results highlight the importance of improving the quality of online information environments—for example, through algorithms that reduce exposure to extreme or harmful content and increase the visibility of constructive materials. Interventions informed by cognitive-behavioral therapy may also help older adults develop critical information appraisal skills, thereby mitigating potential negative effects of internet use on social cognition and health behavior. Furthermore, the analysis revealed gender differences in these mechanisms. Although all genders showed a significant association between internet use and mental health, the relationship was somewhat more pronounced among females. Women may be more affected by social comparison and emotional content online, making them more vulnerable to declines in perceived fairness (67). Multi-group analyses indicated that perceived social fairness played a stronger mediating role among women, while physical activity was a more prominent mediator among men (68, 69). These patterns suggest that gender-specific intervention approaches may be beneficial: for older women, enhancing critical media literacy and cognitive reframing skills may help; for older men, maintaining physical activity through digital and community support may be especially important.

This study has several important limitations that are central to the interpretation of our findings. First and most critically, the cross-sectional design fundamentally limits causal inference. While we have identified significant associations, the temporal sequence among variables cannot be definitively established. This raises the strong possibility of reverse causation—for instance, older adults with better mental health may be more inclined to adopt and use internet technologies, rather than internet use leading to improved psychological wellbeing. Similarly, individuals with more positive psychological states might report higher levels of perceived social fairness regardless of their actual internet exposure. Although we employed propensity score matching to address observable pre-existing differences between internet users and non-users, this method cannot eliminate all sources of unmeasured confounding. Second, the measurement approach presents constraints: our mental health measure was based on a single-item self-report, which may not capture the construct’s full complexity and is particularly vulnerable to recall and social desirability biases. Additionally, several key variables (e.g., perceived social fairness, physical activity) relied on ordinal scales from self-reported questionnaires, introducing potential for common method variance. Third, regarding generalizability, our findings are derived from a specific national context—China’s unique sociocultural environment and rapidly evolving digital ecosystem, where internet access, content regulation, and older adults’ usage patterns may differ significantly from other cultural and political contexts. Thus, the observed relationships and their magnitudes may not directly translate to other populations. Future research should address these limitations through longitudinal or experimental designs, incorporate more comprehensive and objective measures (e.g., digital behavioral traces, device-based physical activity monitoring), and examine whether these mechanisms operate similarly across diverse cultural and digital policy environments.

## Conclusion

Based on survey data from Chinese society, this study systematically investigates the associations between internet use and mental health among older adults. Our results suggest that internet use is not only directly correlated with better mental health outcomes but may also involve complex mediating pathways through perceived social fairness and physical activity. The identified patterns indicate that while internet use might be associated with reduced perceived social fairness (potentially through increased exposure to information and social comparison), which could indirectly negatively influence mental health, it may also correlate with increased physical activity (possibly by enhancing health awareness and social participation), thereby potentially contributing to improved psychological wellbeing. Furthermore, our analyses revealed a statistically supported sequential pathway involving both perceived social fairness and physical activity, suggesting a possible “cognition-behavior” pattern through which internet use might relate to mental health. However, given the cross-sectional nature of our data, these mediating pathways should be interpreted as associational patterns rather than confirmed causal mechanisms. The findings provide preliminary empirical evidence for understanding the complex relationship between digital technology and psychological wellbeing in older adults, highlighting the need for future longitudinal or experimental studies to verify the causality and temporal dynamics of these associations. In light of these associational findings, policy approaches should focus on promoting digital inclusion while cautiously considering interventions that could enhance older adults’ digital literacy, encourage physical activity, and improve the online information environment—all while acknowledging that further evidence is needed to confirm causal efficacy before implementing large-scale policy interventions.

## Data Availability

The original contributions presented in the study are included in the article/supplementary material, further inquiries can be directed to the corresponding author.
